# A Curious Case of Abdominal Pain: Coexisting Left Lower Lobe Pneumonia and Helicobacter pylori-Positive Erosive Gastritis

**DOI:** 10.7759/cureus.99943

**Published:** 2025-12-23

**Authors:** Dipra Dattasarma, Souvik Sen, Sudipta Sardar, Mainak Mandal, Abhishek Chanda

**Affiliations:** 1 Internal Medicine, KPC Medical College and Hospital, Kolkata, IND

**Keywords:** atypical presentation, epigastric pain, erosive gastritis, helicobacter pylori, lower lobe consolidation, pneumonia

## Abstract

Pneumonia typically manifests with fever, productive cough, dyspnea, pleuritic chest pain, and tachypnea; however, atypical or uncommon symptoms may obscure the underlying diagnosis. Lower lobe pneumonias may uncommonly present with abdominal pain, leading clinicians toward gastrointestinal diagnoses before respiratory pathology becomes apparent. We report a case where severe epigastric pain was produced by two independent pathologies, left lower lobe pneumonia and *Helicobacter pylori* (*H. pylori*)-positive erosive gastritis, creating a complex and misleading symptom pattern. This case highlights the importance of a systematic cardiopulmonary examination in all abdominal pain presentations and the risk of anchoring bias when parallel pathologies coexist.

## Introduction

Pneumonia is a common, potentially fatal infection, affecting approximately 450 million people and causing nearly four million deaths annually worldwide, with the highest burden observed in children under five years and older adults [1,2]. In India, community-acquired pneumonia (CAP) occurs at a rate of approximately 5-11 per 1,000 adult person-years and contributes to over one-fifth of the global CAP burden [1]. The disease results from microbial invasion of the distal airspaces, leading to neutrophilic inflammation, alveolar exudation, and consolidation. When the lower lobes are involved, inflammatory irritation of the diaphragmatic pleura may stimulate phrenic nerve afferents (C3-C5), producing referred epigastric or upper abdominal pain that can closely mimic primary gastrointestinal or surgical pathology [1-5].

Gastritis and duodenitis are also highly prevalent worldwide, with an estimated 27.2 million affected individuals in 2021 and projections exceeding 50 million by 2050 [6,7]. Endoscopic studies report erosive gastritis in approximately one-quarter of examined patients, and in many developing countries, *Helicobacter pylori *(*H. pylori*)-associated gastritis affects more than 44% of the population [6,8]. *H. pylori* colonization induces chronic active inflammation, disrupts the gastric mucosal barrier through urease activity and virulence factors, and promotes acid-mediated mucosal injury, resulting in fundal and antral erosions that manifest as epigastric pain, dyspepsia, nausea, or upper gastrointestinal bleeding [6-9].

In our case, the coexistence of lower lobe pneumonia and* H. pylori*-related erosive gastritis created a pathophysiologic convergence in which diaphragmatic pleural irritation and gastric mucosal injury simultaneously generated upper abdominal pain, thereby obscuring the primary etiology. The differential diagnosis in such presentations is broad and includes peptic ulcer disease, erosive gastritis, acute pancreatitis, biliary or hepatobiliary pathology, mesenteric ischemia, bowel obstruction, renal colic, myocardial ischemia, aortic dissection, subdiaphragmatic abscess, and lower lobe pneumonia [1-5,10]. Optimal management requires targeted antimicrobial therapy and supportive care for pneumonia, guided by disease severity and local resistance patterns, alongside proton pump inhibitor-based* H. pylori* eradication regimens, mucosal protectants, and dietary modification for erosive gastritis [6-9].

This case underscores how overlapping pathophysiological mechanisms in two common diseases can generate an atypical clinical presentation, necessitating systematic multisystem evaluation and vigilance against anchoring bias in patients presenting with abdominal pain [10]. It also represents an uncommon presentation of a common disease compounded by a second pathology. In the current era of increasing recognition of rare diseases due to advanced diagnostic capabilities, clinicians may focus disproportionately on common presentations of uncommon conditions; however, uncommon presentations of common diseases must not be overlooked.

## Case presentation

A 43-year-old male patient with no significant comorbidities presented with intermittent mild upper abdominal discomfort for the past four months, which had acutely worsened over the preceding 24 hours. During this period, he developed fever, two episodes of nonbilious vomiting, and severe generalized weakness. On initial evaluation, he appeared dehydrated with a dry tongue. Abdominal examination demonstrated left hypochondriac tenderness. An ultrasonography of the whole abdomen performed outside the hospital revealed no abnormalities.

At presentation to the emergency department, in the absence of respiratory symptoms such as cough or dyspnea, a provisional diagnosis of acute gastroenteritis was made, and the patient was admitted to the general ward. ECG was within normal limits.

In the ward, his vital parameters were stable (pulse, 94/min; BP, 110/70 mmHg; temperature, 97.9°F; capillary blood glucose, 137 mg/dL). Abdominal examination continued to show marked left hypochondriac tenderness without guarding or rigidity. Cardiopulmonary examination, however, revealed left lower lobe crepitations.

Laboratory evaluation indicated microcytic iron deficiency anemia, CRP of 12 mg/L, and procalcitonin of 5.5 ng/ml, suggesting some severe infection such as sepsis and hypokalemia (all the laboratory findings are compiled in Table 1). The patient was initially treated with intravenous fluids with potassium chloride (KCL) injection, paracetamol for fever, proton pump inhibitor to prevent stress-induced gastric ulcer, drotaverine injection for abdominal pain and empirical antibiotic ceftriaxone (for severe infection). Echocardiography demonstrated normal cardiac structure with an ejection fraction of 67%. However, none explained the abdominal pain properly.

**Table 1:** Laboratory findings with reference range AST: aspartate aminotransferase; SGOT: serum glutamic-oxaloacetic transaminase; ALT: alanine aminotransferase; SGPT: serum glutamic-pyruvic transaminase; ALP: alkaline phosphatase; TSH: thyroid-stimulating hormone; HCV: hepatitis C virus; HIV: human immunodeficiency virus Lab findings indicate iron deficiency anemia, severe infection, sepsis, hypokalemia

Parameter	Value	Reference range
Capillary blood glucose	137 mg/dL	70-140 mg/dL random
Hemoglobin	10.1 g/dL	13-17 g/dL (male)
Total leukocyte count	8,700/µL	4,000-11,000/µL
Mean corpuscular volume (MCV)	73 fL	80-96 fL
Platelet count	1.6 lakh/µL	1.5-4.5 lakh/µL
Serum amylase	26 U/L	30-110 U/L
Serum lipase	18 U/L	0-160 U/L
C-reactive protein (CRP)	12 mg/L	<5 mg/L
Procalcitonin	5.5 ng/mL	<0.1 ng/mL
Bilirubin total	1.1 mg/dL	0.1-1.2 mg/dL
Bilirubin conjugate	0.2 mg/dL	<0.3 mg/dL
AST (SGOT)	43 U/L	5-40 U/L
ALT (SGPT)	46 U/L	5-41 U/L
ALP	83 U/L	40-130 U/L
Serum creatinine	1.3 mg/dL	0.7-1.3 mg/dL
Blood urea	18 mg/dL	10-50 mg/dL
Sodium	143 mmol/L	135-145 mmol/L
Potassium	2.8 mmol/L	3.5-5.0 mmol/L
TSH	1.19 µIU/mL	0.36-5.6 µIU/mL
Free T4	1.21 ng/dL	0.8-2.0 ng/dL
Urine routine	Normal	-
Urine culture	No growth	No growth
Viral markers (HBsAg, anti-HCV, anti-HIV	Nonreactive	Nonreactive

**Figure 1:**
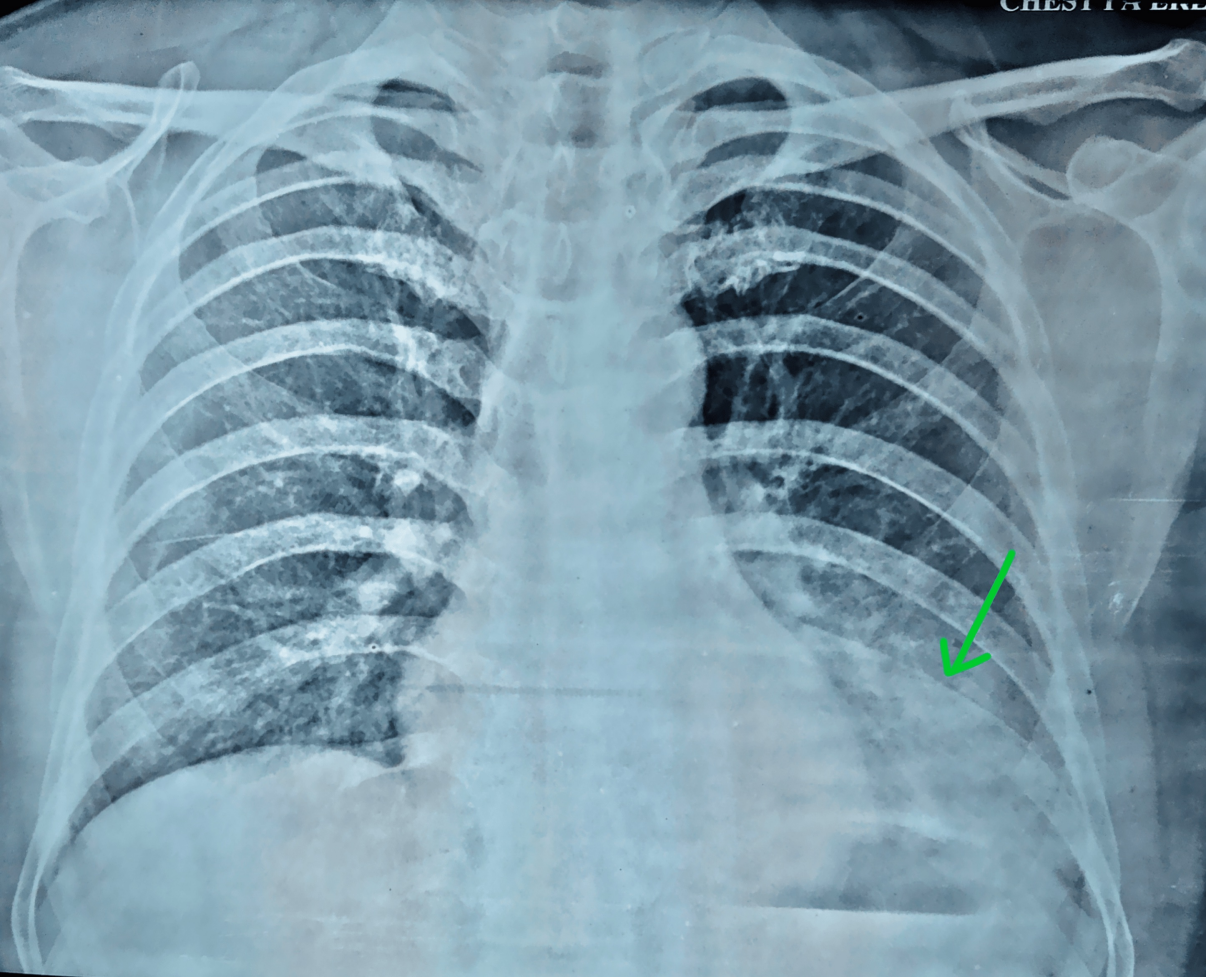
The chest radiograph reveals a left basal pulmonary consolidation (green arrow)

By day 2, the patient remained febrile with persistent epigastric pain. Repeat auscultation again revealed left basal crepitations. A chest radiograph showed left basal consolidation (Figure 1).

**Figure 2:**
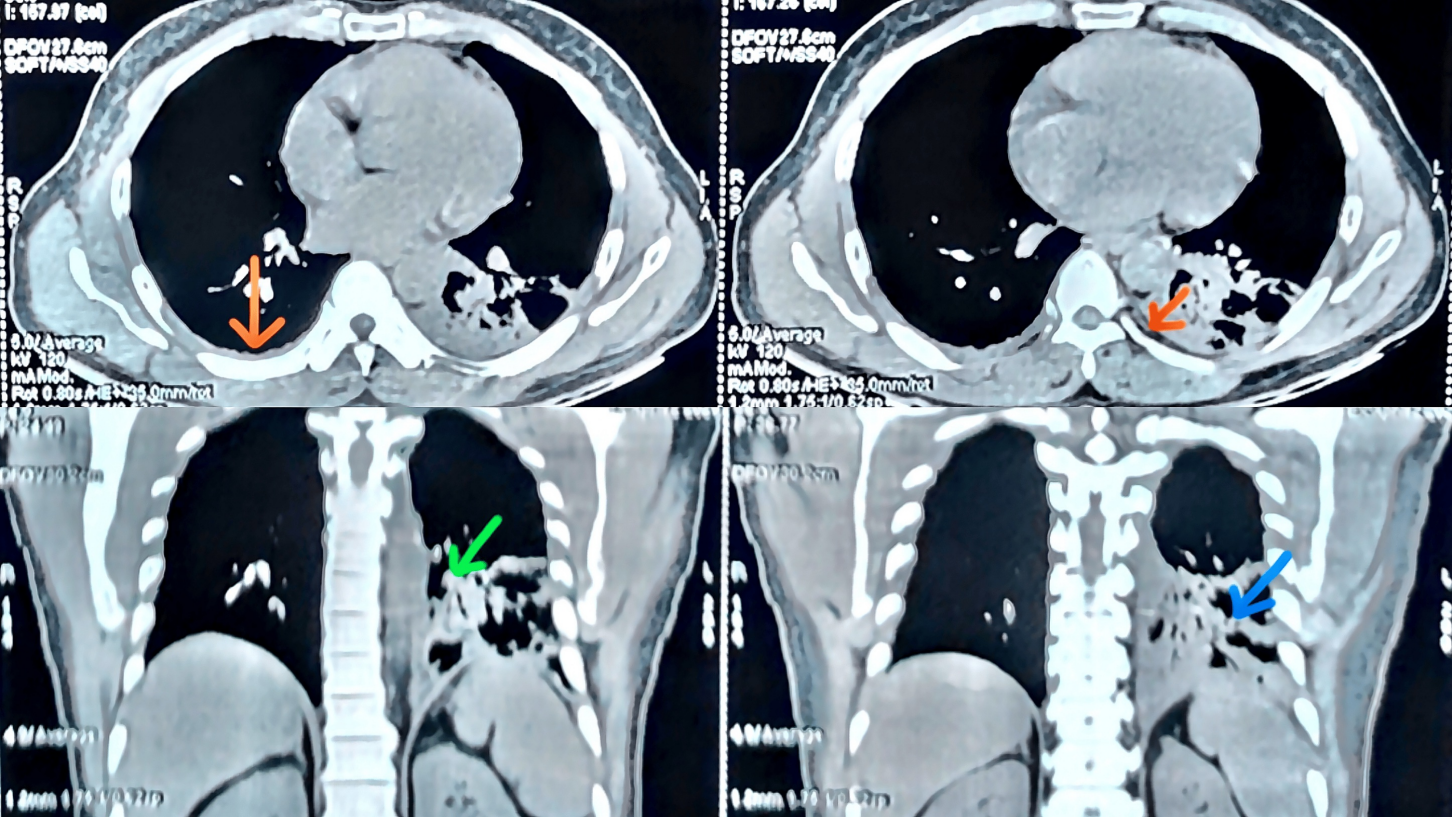
The HRCT thorax image HRCT: high-resolution computed tomography The HRCT thorax demonstrates extensive patchy consolidation in the left lower lobe (blue arrow). Additionally, there is mediastinal and left hilar lymphadenopathy (green arrow), with bilateral minimal posterior basal pleural thickening (orange arrow)

A contrast-enhanced CT scan of the abdomen revealed no acute intra-abdominal pathology apart from incidental benign prostatic hyperplasia. High-resolution CT of the thorax confirmed extensive patchy pneumonitis involving the left lower lobe without pleural effusion (Figure 2). Intravenous piperacillin-tazobactam (for broad spectrum coverage against common community acquired and Gram-negative pathogens, including *Streptococcus pneumoniae*, *Haemophilus influenzae,* and *Enterobacteriaceae*) and doxycycline ( for atypical organisms, like *Mycoplasma pneumoniae*, *Legionella *species, etc. ) were initiated, resulting in gradual defervescence.

**Figure 3:**
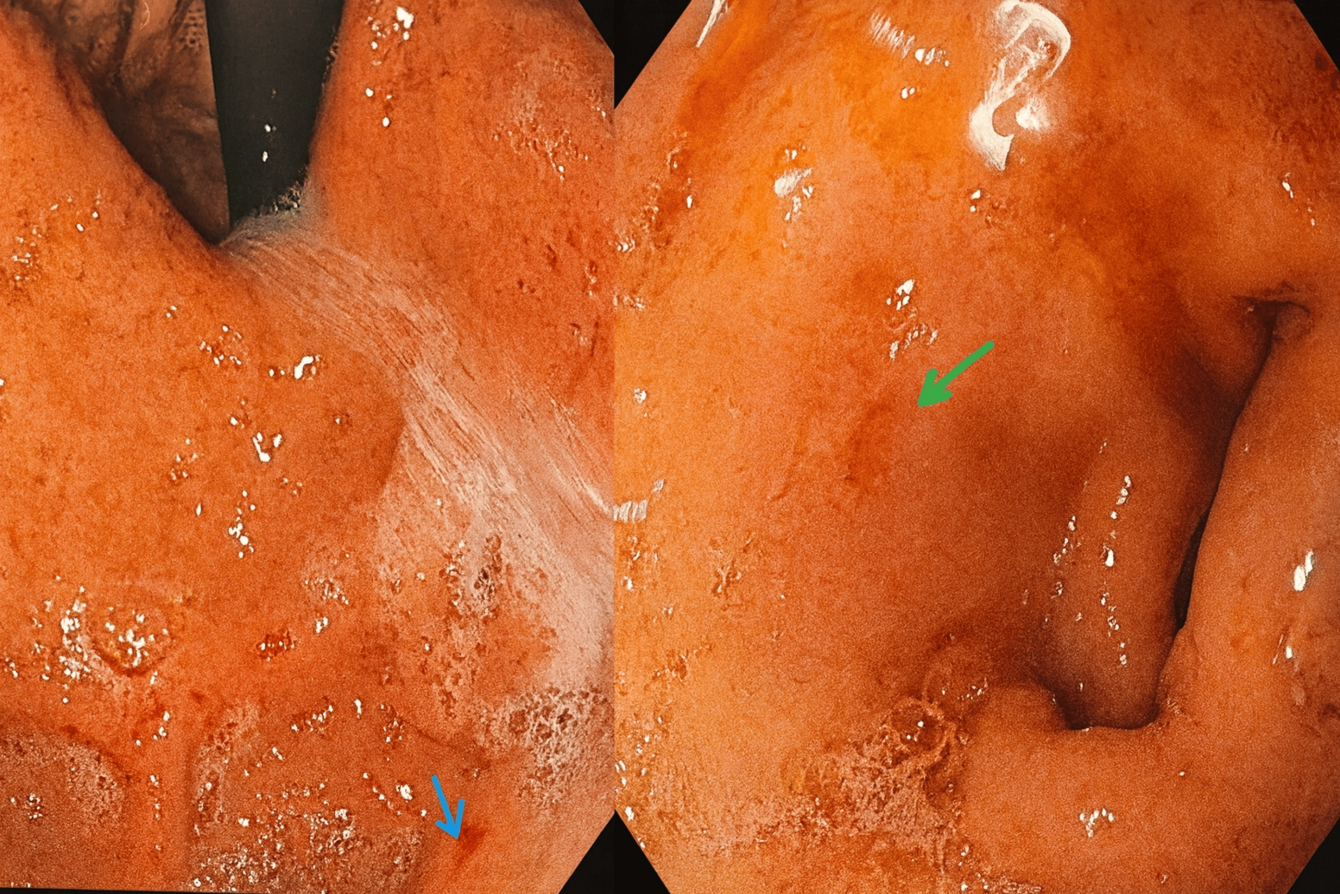
The upper GI endoscopy image The upper GI endoscopy demonstrated multiple erosions in the fundus and antrum consistent with erosive gastritis (blue arrow), along with gastric antral erythema (green arrow). The rapid urease test was positive, supporting *H. pylori-*associated mucosal injury

The patient became afebrile by day 4, with a reduction in lower lobe crepitations; however, left hypochondriac pain, though reduced, persisted. Upper gastrointestinal endoscopy performed on day 7 revealed multiple fundal and antral erosions (Figure 3), with a rapid urease test positive for *H. pylori*. The duodenum appeared normal, and no ulcer crater or active bleeding was observed.

**Figure 4:**
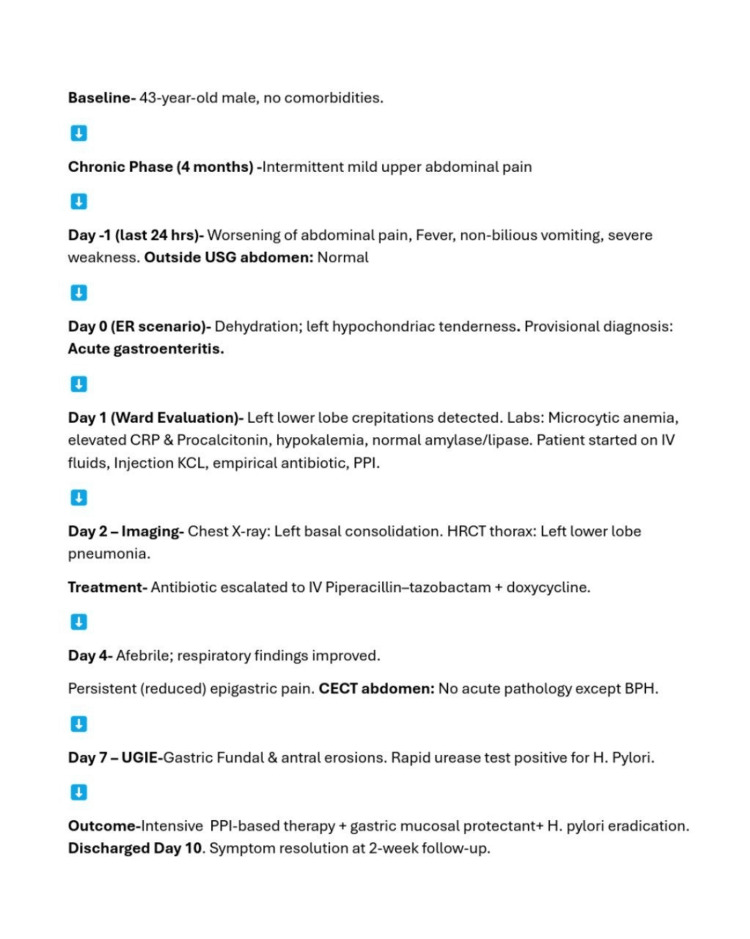
Chronology of the events and their management

The patient was started on twice daily dose of proton pump inhibitors (for gastric acid suppression), mucosal protectants (sucralfate for cytoprotective effect), and dietary modifications. His abdominal pain progressively improved, and he was discharged on day 10 with *H. pylori* eradication therapy, bismuth-based quadruple therapy (because in Indian subcontinent, there is increased clarithromycin resistance). At two-week follow-up, his symptoms had markedly resolved, and he remained clinically stable. The following figure (Figure 4) depicts the chronology of the events and their management.

## Discussion

This case demonstrates the diagnostic complexity that arises when two distinct pathological processes produce overlapping symptoms. Lower lobe pneumonia is known to mimic acute abdomen due to diaphragmatic irritation and phrenic nerve-mediated referred pain [1-5]. Patients may exhibit minimal or no respiratory symptoms early in the course, leading clinicians toward gastrointestinal or surgical causes.

In this patient, the presence of persistent epigastric pain despite the initiation of appropriate antibiotics prompted further investigation, revealing *H. pylori*-positive erosive gastritis. Gastric erosions alone can produce significant upper abdominal pain [6,7]. Their coexistence with lower lobe pneumonia created a compounded symptom pattern where one pathology masked and amplified the other. Prior literature also discusses potential interactions between *H. pylori*-associated gastric disease and pulmonary pathology [8,9], though direct dual presentations like this remain rare. This case highlights several key learning points: A systematic cardiopulmonary examination is essential in all patients with abdominal pain, particularly when fever is present [1-5,10]. Lower lobe pneumonia must remain a differential diagnosis for unexplained upper abdominal pain [1-4]. Anchoring bias may occur when early findings (e.g., gastritis) distract from underlying parallel disease processes [10]. Persistent or disproportionate abdominal pain warrants further evaluation even when an initial diagnosis seems adequate [8,9].

## Conclusions

The coexistence of left lower lobe pneumonia and *H. pylori*-associated erosive gastritis can create a diagnostically challenging presentation, as both conditions independently generate upper abdominal pain through distinct mechanisms. In this patient, diaphragmatic irritation from lower lobe pneumonia and mucosal inflammation from erosive gastritis produced overlapping symptoms that initially obscured the underlying pulmonary pathology. This underscores the importance of avoiding diagnostic anchoring, particularly when clinical evolution is atypical or inadequately explained by a single diagnosis. A structured, multisystem approach, including careful cardiopulmonary examination and timely imaging, remains essential when abdominal pain coexists with fever, even in the absence of respiratory symptoms. Importantly, this case highlights the need for general practitioners to broaden their clinical perspective, remain alert to uncommon manifestations of common diseases, and recognize subtle but significant deviations from typical presentations. Such vigilance can prevent diagnostic delays and reduce the risk of potentially serious complications.
